# Towards Optimized Awake Bruxism Metrics: Empirical Definition of Minimal Ecological Momentary Assessment Requirements

**DOI:** 10.1111/joor.70263

**Published:** 2026-07-09

**Authors:** Alona Emodi‐Perlman, Lihi Keren, Anna‐Yael Czygrinow, Khalil Masarwe, Noa Ventura, Ilana Eli

**Affiliations:** ^1^ Department of Oral Rehabilitation, the Maurice and Gabriela Goldschleger School of Dental Medicine Grey Faculty of Medicine and Health Sciences, Tel‐Aviv University Tel‐ Aviv Israel

**Keywords:** awake bruxism, ecological momentary assessment, EMA, smartphone application

## Abstract

**Background:**

Ecological Momentary Assessment (EMA) is increasingly used to assess awake bruxism (AB) in real time. However, current protocols typically require 7 days of monitoring and high daily response rates, potentially limiting compliance.

**Objectives:**

To empirically establish the shortest monitoring duration and daily response threshold that still yield reliable estimates of AB behaviours through smartphone‐based EMA.

**Methods:**

Data from three EMA studies were pooled, yielding 327 participants who completed smartphone‐based monitoring. Five core AB behaviours (relaxed jaw muscles, bracing, teeth contact, clenching, and grinding) were analysed. Temporal stability was evaluated using intraclass correlation coefficients (ICC[A,1]) by comparing progressively shorter monitoring windows with the complete 7‐day dataset. Compliance‐threshold analyses examined the relationship between minimum daily responses, reliability, and participant retention.

**Results:**

The pooled dataset included over 32 000 EMA reports. Relaxed jaw condition was the most frequently reported behaviour (52.2%), followed by teeth contact (18.6%) and bracing (14.5%). Clenching (6.9%) and grinding (1.4%) were less frequent. Four of five behaviours achieved excellent stability after three monitoring days (ICC ≥ 0.86), whereas grinding reached stability after 2 days (ICC = 0.81). Increasing the minimum daily response threshold improved reliability but substantially reduced sample retention. A threshold of ≥ 12 answered alerts/day provided the best balance between reliability and retention.

**Conclusions:**

Reliable assessment of the five core awake bruxism behaviours can be achieved using a 3‐day EMA protocol with at least 12 answered alerts/day. These findings support a reduction in monitoring burden while maintaining data quality.

## Graphical abstract

A pooled analysis of 327 participants and more than 32 000 EMA reports identified the minimum monitoring burden required for reliable awake bruxism assessment. Four of five core behaviours reached stable estimates after 3 monitoring days, while grinding stabilized after 2 days. A threshold of ≥ 12 completed alerts/day provided the optimal balance between reliability and participant retention, supporting shorter and more feasible EMA protocols for research and clinical applications.

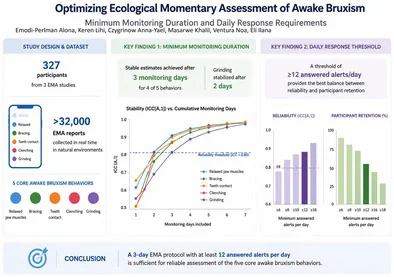

## 1 Introduction

Awake bruxism (AB) is a wakeful masticatory muscle activity involving repetitive or sustained tooth contact and/or bracing or thrusting of the mandible [1, 2], with a prevalence of 16%–30% [3, 4, 5]. AB is considered a secondary phenomenon (an indicator of underlying processes) and may act as a risk factor, protective factor, or neutral factor depending on the individual's clinical context [1, 6].

The Standardized Tool for the Assessment of Bruxism (STAB) [7] has organized assessment into three domains system: (i) subject‐based assessment; (ii) clinically based assessment; and (iii) instrumentally based assessment (including electromyography and ecological momentary assessment ‐EMA) [1, 2].

An introduction of EMA through a smartphone application provides the ability to collect real‐time self‐reporting of AB in natural environments, capturing behaviour frequency as they happen. Within the framework of EMA, awake‐bruxism behaviour is captured through self‐reports provided at discrete alert‐based time points. This approach offers valuable real‐time ecological data but does not quantify the true frequency or temporal dynamics of AB events occurring between prompts, thereby providing a sampled rather than continuous behavioural record. These methodological constraints highlight the need for validation studies integrating EMA with simultaneous electromyographic recordings to determine the reliability and accuracy of EMA‐derived measures. Additionally, prompting patients throughout the day builds self‐awareness and may act as a behavioural intervention [8, 9, 10]. To date, most published smartphone‐based EMA studies investigating AB behaviours have used the BruxApp platform (BruxApp, WMA Srl., Florence, Italy) [8].

Originally, the BruxApp tracked five key awake bruxism behaviours (grinding, clenching, teeth contact, jaw bracing, and relaxed jaw muscles) [11, 12]. Studies that employed this version (BruxApp version 1) showed that a combination of single point self‐report and EMA increased the ability to assess AB [13, 14, 15]. The five‐behaviour version was also shown as useful to monitor AB over time and increase individuals' awareness, particularly to the behaviours of bracing and clenching [16, 17]. A later version of the BruxApp increased the number of reported behaviours to ten, adding five nonfunctional awake oral behaviours (BruxApp version 2) [18, 19].

In spite of its advantages, major limitations of EMA relate to compliance and comprehension. Nykänen et al. [20] highlighted that lay subjects often have poor initial understanding of AB terms. Saracutu et al. and Colonna et al. [15, 21] showed that a minimum response rate of 12 out of 20 daily alerts is required for daily monitoring to be considered valid. An initial effort to standardize future EMA reporting [21] found that participants responded to about 70% of smartphone alerts on average and required about 10 days to reach the target of 7 days.

Many EMA studies currently employ a 7‐day protocol, despite the absence of empirical evidence supporting it. Completing a 7‐day schedule that includes 12 behavioural prompts per day is demanding and often leads to considerable attrition, with attrition rates ranging from one‐fifth to one‐third of enrolled participants [13, 19, 22]. Whereas prior Ecological Momentary Assessment studies emphasized prevalence, questionnaire comparisons, or links to temporomandibular disorders (TMD), the present study focuses on determining the minimal monitoring needed. Its aim was to optimize EMA methodology for assessing AB by identifying the minimum number of monitoring days and daily reports needed to obtain reliable and stable estimates of AB behaviour frequency. This may help unlock the full potential of EMA in AB assessment by increasing the feasibility and accessibility of clinical and research protocols, thereby enhancing long‐term participant engagement and compliance.

*(i) A limited number of monitoring days will yield stable estimates of the five core awake bruxism behaviours; and (ii) an empirical daily compliance threshold that balances reliability with participant retention can be identified*.

### 1.1 Clinical Relevance

Excessive EMA prompting can erode engagement and weaken data quality. Identifying the minimal effective sampling strategy supports reliable assessment with improved feasibility for real‐world implementation.

## 2 Material and Methods

### 2.1 Population

The study was conducted as a pooled secondary analysis of three previously completed observational EMA investigations of awake bruxism, all of which employed a smartphone‐based assessment protocol in samples drawn from the general population. The pooled sample was composed of three distinct populations as follows:

1. Cohort No. 1 (S1): Data describing the S1 cohort have been presented previously [13]. In brief, 147 subjects downloaded the application of whom 106 completed 7 days of a full valid report (28% attrition rate). Mean age of subjects included in the analysis was 24.4 ± 2.99, years, 63% were female. Subjects reported five AB behaviours as follows (BruxApp version 1): (i) Relaxed jaw muscles; (ii) Bracing; (iii) Teeth contact; (iv) Clenching; and (v) Grinding.

2. Cohort No. 2 (S2): Data describing the S2 cohort have been presented previously [17]. 150 subjects downloaded the EMA application of whom 118 participants completed 7 days of a full valid report of ten behaviours as described below (21.3% attrition rate). Mean age of subjects included in the analysis was 28.6 ± 3.6 years, 62.7% were female. The EMA version in the S2 cohort required the report of ten reported behaviours (BruxApp version 2): five similar to the one described in S1 and five additional ones as follows: (vi) Tongue forcibly pressing against the teeth (push tongue); (vii) Tongue positioned between the teeth (place tongue between teeth); (viii) Biting/chewing on tongue, lips or cheeks (bite/chew/play with tongue/checks/lips); (ix) Holding or biting objects between the teeth (hold/bite objects); and (x) Chewing gum.

3. Cohort No. 3 (S3): The EMA version in the S3 study required the report of ten reported behaviours as described above (BruxApp version 2). The S3 sample was drawn from a cohort distinct from those in S1 and S2; all participants had previously completed a cross‐sectional online survey, the findings of which have already been published [23]. Individuals who took part in the survey and provided consent to be contacted for a follow‐up study using the EMA application were subsequently invited to participate. 144 subjects downloaded the EMA application of whom 103 completed 7 days of a full valid report of ten behaviours (28% attrition rate). Mean age of subjects included in the analysis was 30.5 ± 9.1 years, 63.0% were female. All participants reported ten AB behaviours, like the S2 cohort.

The total number of participants in the pooled sample was 327. Each one of the three studies was approved by the ethical committee of Tel‐Aviv University, approval Nos. 000693‐1 (for S1), 0006074–2 (for S2), and 0009166–3 (for S3). Written informed consent was obtained from all the participants.

### 2.2 Instruments

EMA was performed with a designated smartphone application (BruxApp, WMA Srl., Florence, Italy) as explained in former publications [12, 13, 18]. In brief, participants received explanation/training sessions on how to use the application and how to recognize the different behaviours and downloaded the application to their smartphones. The application sent each participant 20 alert sounds at random hours during the day. At each alert sound, the subject was requested to indicate on his/her smartphone the present condition of his/her teeth and jaw condition. There were 5 possibilities in the first study (S1, see above) and 10 possibilities in the following two studies (S2 and S3, see above). Data was reported over a seven‐day period. Days with less than 12 responded alerts were discarded, and the application continued until 7 complete days were reported. Subjects who failed to complete 7 days of valid responses were not included in the study.

### 2.3 Statistics

To correct for variability in daily compliance, the primary outcome for each behaviour on each monitoring day was the daily behaviour percentage. Because S1 presented five behavioural response options per alert and S2/S3 presented ten, the denominator was reconstructed separately per study: for S1, the denominator equalled the sum of the five shared behaviour counts on a given day; for S2 and S3, it equalled the sum of all ten behaviour counts.

Temporal stability was quantified using the intraclass correlation coefficient for absolute agreement, single measures [ICC(A,1)] [24, 25]. To determine the minimum number of monitoring days required for a stable behavioural estimate, ICC(A,1) for each behaviour was computed using progressively longer cumulative windows. For window length L (L = 1 to 7), the L‐day estimate was the mean of the daily behaviour percentages across days 1 through L; the gold standard was the mean across all 7 days. ICC(A,1) was computed between these two vectors across participants for all 327 participants for the five original behaviours. The minimum stable monitoring duration was defined as the smallest L for which ICC(A,1) ≥ 0.80. To characterize agreement at this threshold, the Pearson correlation (r) and the mean absolute error (MAE) were also calculated.

Each ICC(A,1) estimate was accompanied by a 95% confidence interval (CI). Sensitivity analyses were conducted separately in the S1 cohort and in the combined S2 + S3 cohort to evaluate whether stabilization patterns were consistent across app versions and reporting formats.

Compliance‐threshold analysis, accounting for both reliability gains and sample retention, was used to define the minimum number of alerts to be answered per day.

Continuous variables were reported as means and standard deviations and medians with interquartile ranges where distributions were non‐normal.

All analyses were conducted in R (version 4.3.x; R Core Team, 2024) with set. Seed (12345) to ensure reproducibility.

## 3 Results

The pooled sample comprised 327 participants (mean age 30.5 ± 9.1 years, 63.0% female) recruited across the three datasets (S1, S2, S3).

The total number of responded alerts was 32 675.

Mean daily answered alerts across the 7‐day period were 15.0 ± 1.9 for S1, 13.2 ± 0.9 for S2, and 14.7 ± 1.8 for S3, corresponding to a pooled mean of 14.3 ± 1.8 answered alerts per day.

Analysis of the five AB behaviours (relaxed muscles; bracing; teeth contact; clenching, grinding) showed that relaxed jaw condition was the most frequently reported (52.15% ± 26.11%), teeth contact (18.61% ± 14.34%) and jaw bracing (14.52% ± 15.25%) were the next most common behaviours. Clenching was reported less frequently (6.92% ± 10.57%), and grinding was rare (1.36% ± 3.92%). The high standard deviations relative to the means for clenching and grinding indicate substantial between‐person heterogeneity and right‐skewed distributions.

Relaxed jaw posture showed a modest increase from day 1 (48.9%) to day 7 (54.9%). All other behaviours remained within a range of approximately 3 percentage points across days.

Analysis of the ten behaviour reports included in S2 and S3 revealed that the five initial behaviours (relaxed muscles; bracing; teeth contact; clenching, grinding) showed a pattern similar to the S1 cohort that assessed only five behaviours. The five additional behaviours (push tongue, place tongue between teeth, bite/chew/play with tongue/checks/lips, hold/bite objects and chew gum) showed a mean frequency of less than 2% (Figure 1). The near‐complete overlap of the two profiles indicates that four of the five original awake bruxism behaviours account for most reported oral behavioural observations. The additional behaviours occurred at very low frequencies and accounted for a small proportion of all EMA responses and therefore had limited influence on overall behavioural distribution.

**FIGURE 1:**
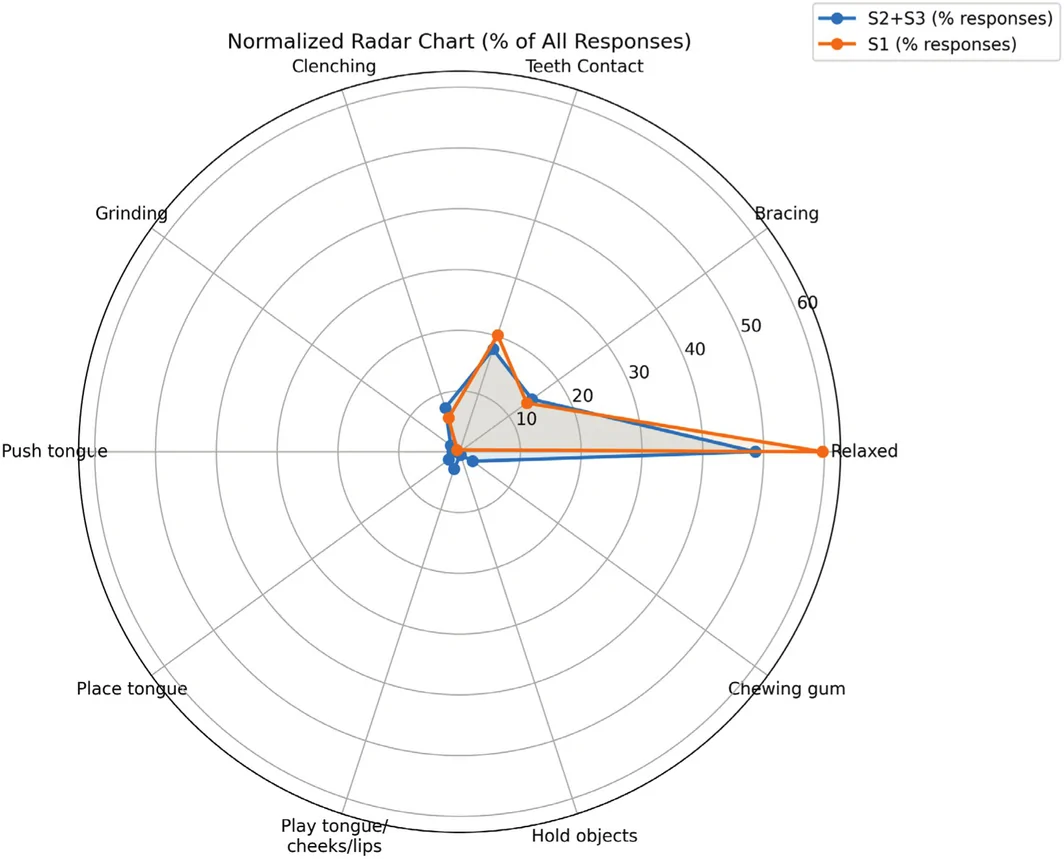
Overlay of pooled 5‐behaviour profile (S1, *N* = 106) and 10‐behaviour profile (S2 ± S3, *N* = 221) *. *The chart displays mean percentage frequency of each behaviour as recorded by EMA. Orange line represents the five‐behaviour profile from S1 (Relaxed jaw, Bracing, Teeth contact, Clenching, Grinding). The Blue line represents the pooled ten‐behaviour profile from cohorts S2 and S3 (which includes the five core behaviours plus Push tongue, Place tongue between teeth, Bite/chew/play with tongue/checks/lips, Hold/bite objects and Chew gum).

Due to resembling distribution patterns in behaviour frequencies, and the very low occurrence of the five additional behaviours introduced in S2 and S3, only the original five behaviours are discussed here. Detailed means for all ten oral behaviours recorded in S2 and S3 are provided in the (Table S1).

### 3.1 Monitoring‐Day Stability

ICC values of the five core behaviours (relaxed muscles; bracing; teeth contact; clenching, grinding) increased monotonically from day 1 to day 7. At a single day (*L* = 1), ICC values ranged from 0.490 (clenching) to 0.654 (bracing), indicating poor to moderate reliability of single‐day estimates. ICC values improved substantially with the addition of a second day for all behaviours, particularly for teeth contact (0.500 to 0.756) and clenching (0.490 to 0.775), reflecting that these behaviours show high day‐to‐day within‐person variability that is effectively averaged out after 2 days. The ICC ≥ 0.80 stability threshold was reached after three monitoring days for four of the five behaviours: relaxed jaw posture (ICC = 0.885), bracing (ICC = 0.860), teeth contact (ICC = 0.874), and clenching (ICC = 0.887). Tooth grinding reached this threshold after two monitoring days (ICC = 0.806), consistent with its near‐zero median and the fact that even a small number of observations is sufficient to identify the predominant pattern of non‐occurrence or rare occurrence in most participants (Figure 2).

**FIGURE 2:**
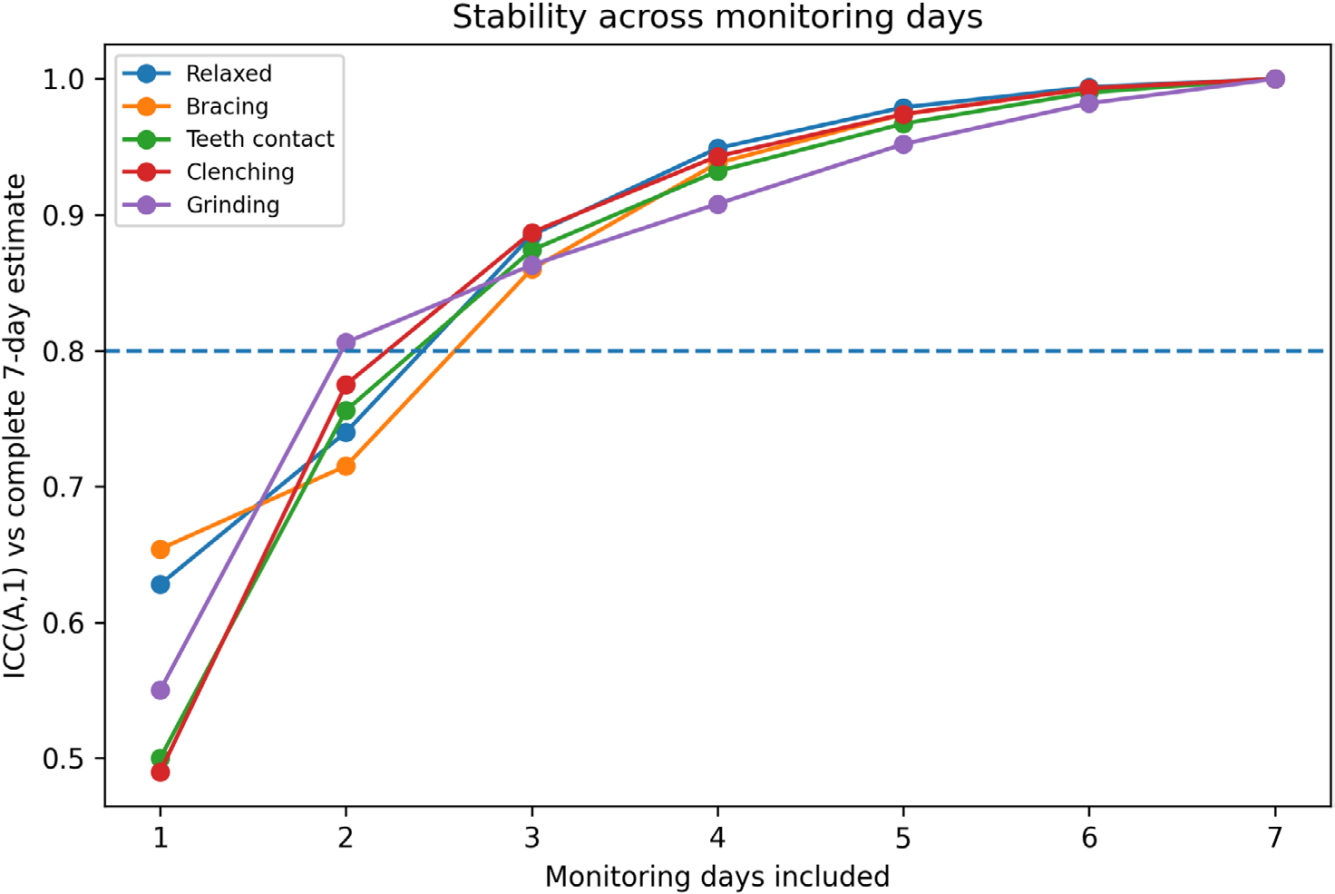
ICC(A,1) as a function of cumulative monitoring days for five oral behaviours (*N* = 327).

Detailed ICC values by cumulative monitoring window are presented in the (Table S2).

Pearson correlations at the minimum stable day ranged from *r* = 0.812 (grinding, *L* = 2) to *r* = 0.889 (relaxed jaw posture, *L* = 3), confirming strong linear agreement between shortened and complete estimates. MAE values ranged from 0.98 percentage points for grinding to 9.07 percentage points for relaxed jaw posture, indicating that the 3‐day estimate of relaxed jaw posture deviates from the 7‐day value by approximately 9 percentage points on average, a magnitude that is clinically acceptable given the broad behavioural range (0–100%) and the high ICC. Pearson correlation, ICC(A,1), and mean absolute error (MAE) between the *L*‐day estimate and the 7‐day gold standard at each behaviour's stability threshold are presented in Table 1.

**TABLE 1:** Agreement statistics at the minimum stable monitoring duration.

Behaviour	Min. days (L)	ICC(A,1) (95% CI)	Pearson *r*	MAE (pp)	*N*
Relaxed jaw posture	3	0.885 (0.853–0.909)	0.889	9.07	327
Jaw bracing	3	0.860 (0.815–0.894)	0.861	5.35	327
Teeth contact	3	0.874 (0.840–0.900)	0.876	5.09	327
Clenching	3	0.887 (0.841–0.925)	0.888	2.64	327
Grinding	2	0.806 (0.606–0.903)	0.812	0.98	327

*Note:* ICC(A,1) = intraclass correlation coefficient (two‐way random, absolute agreement). *r* = Pearson correlation between L‐day mean and 7‐day gold standard. MAE = mean absolute error in percentage‐point units. pp = percentage points. CI = 95% confidence interval.

Sensitivity analyses performed separately for the S1 cohort (5 behaviours) and a pooled S2 + S3 cohort (10 behaviours) yielded highly similar stabilization patterns for four of the five core awake bruxism behaviours supporting the robustness of the shortened EMA protocol. Small cohort‐specific differences were observed for some behaviours, but the overall conclusion that a 3‐day monitoring window captures the five core behaviours remained supported. Cohort‐specific sensitivity analysis is presented in the (Table S3).

### 3.2 Compliance‐Threshold Analysis

Across all five awake bruxism behaviours, reliability improved as the minimum number of answered alerts per day increased. At the minimum stable monitoring duration (L = 2–3 days), ICC(A,1) values were already in the good range at the lowest compliance threshold (T = 1), with ICCs between 0.806 and 0.887. Increasing the daily compliance threshold produced progressively higher reliability estimates, reaching excellent levels (ICC ≥ 0.90) for most behaviours at T ≥ 12. This improvement, however, was accompanied by a reduction in sample size, which decreased from approximately 327 participants at T = 1 to about 86 participants at T = 12, and to fewer than 50 participants at T = 16. The recommended threshold of T ≥ 12 reflects the optimal balance between reliability and participant retention: ICCs consistently reached excellent levels for all behaviours, while maintaining a sufficiently large sample for stable estimation.

Detailed ICC(A,1) values at each compliance threshold T (minimum answered alerts per day) for each behaviour at its minimum stable monitoring duration are presented in Table 2.

**TABLE 2:** ICC(A,1) and sample size at each compliance threshold, at minimum stable monitoring duration.

Behaviour	L	T = 1 *n*/ICC (95% CI)	TT = 8 *n*/ICC (95% CI)	T = 10 *n*/ICC (95% CI)	T = 12 *n*/ICC (95% CI)	T = 14 *n*/ICC (95% CI)	T = 16 *n*/ICC (95% CI)
Relaxed jaw posture	3	327/0.885 (0.854–0.909)	313/0.895 (0.868–0.919)	299/0.904 (0.878–0.924)	**279/0.911** **(0.886–0.930)**	86/0.954 (0.926–0.972)	22/0.960 (0.892–0.990)
Jaw bracing	3	327/0.860 (0.811–0.895)	313/0.869 (0.823–0.902)	299/0.885 (0.851–0.914)	**279/0.895** **(0.859–0.921)**	86/0.951 (0.898–0.974)	22/0.899 (0.772–0.965)
Teeth contact	3	327/0.874 (0.840–0.901)	313/0.877 (0.842–0.903)	299/0.881 (0.848–0.907)	**279/0.880** **(0.846–0.908)**	86/0.900 (0.858–0.933)	22/0.877 (0.744–0.945)
Clenching	3	327/0.887 (0.844–0.926)	313/0.890 (0.844–0.927)	299/0.891 (0.849–0.927)	**279/0.888** **(0.841–0.928)**	86/0.939 (0.911–0.955)	22/0.964 (0.917–0.987)
Grinding	2	327/0.806 (0.611–0.907)	314/0.807 (0.589–0.907)	300/0.810 (0.602–0.913)	**281/0.811** **(0.594–0.909)**	130/0.917 (0.715–0.962)	44/0.904 (0.606–0.967)

*Note:* L = minimum stable monitoring days. T = minimum answered alerts per day required for inclusion. *n* = number of participants meeting the compliance criterion. ICC = ICC(A,1) in the compliant subsample. Values are *n*/ICC(A,1) (95% CI). Bold = recommended threshold (T ≥ 12).

## 4 Discussion

Defining the minimal effective EMA protocol can facilitate practical clinical and research implementation, ensure high‐quality data while reducing the monitoring load for subjects with complex clinical profiles.

The present investigation is the first study to empirically determine the minimum monitoring burden required for reliable EMA‐based assessment of awake bruxism. Previous studies adopted seven‐day monitoring protocols largely by convention. In the pooled sample of 327 participants, reliable estimates of awake oral behaviours were achieved within a relatively short EMA monitoring window. Analysis of over 32 000 EMA entries showed that a cumulative average based on 3 monitoring days provided acceptable agreement with the full 7‐day estimate. This demonstrates that stable estimates can be obtained after only 3 days, thereby reducing participant burden by 57% (from 7 to 3 days) while maintaining acceptable reliability. A 7‐day EMA protocol, particularly in epidemiological settings, may also introduce bias by inadvertently functioning as an Ecological Momentary Intervention (EMI). The repeated prompts and heightened self‐monitoring inherent to EMA can promote behavioural awareness and modification, thereby blurring the distinction between assessment and intervention effects.

A recent study showed good experience with the EMA smartphone app which included the report of the five core behaviours [26]. Users were primarily motivated by the opportunity to monitor their own AB behaviours, while the limited time allowed to respond to alerts was viewed as the main drawback. It is important to note, however, that the sample studied consisted of a relatively small group of patients referred to an Orofacial Pain Unit, who may have been particularly motivated to use the application as part of managing their painful condition [26]. In the general population, motivation to complete seven consecutive days of EMA reporting is likely to be lower. Indeed, studies using EMA applications in community samples consistently report substantial attrition across the full 7‐day protocol. For example, attrition rates in the S1, S2, and S3 cohorts were 28%, 22%, and 28%, respectively; while other studies have documented attrition rates of up to 33% [22].

Whereas most behaviours remained stable over the 7‐day recording period (within a 3‐percentage‐point range), relaxed jaw increased modestly over the week, from 48.9% to 54.9%. This aligns with previous findings showing that among individuals with TMD‐related pain, the frequency of the relaxed‐jaw behaviour increased significantly from Day 1 to Day 7 [19]. This pattern suggests that passive EMA monitoring may gradually shift into an active form of Ecological Momentary Intervention (EMI), functioning as a biofeedback mechanism that enhances participants' ability to relax their jaw muscles. Such an effect may have direct therapeutic relevance for the management of awake bruxism associated with TMD.

Consistent with previous studies [8], the most prevalent behaviours after relaxed jaw condition were teeth contact, bracing, and clenching. The five additional behaviours introduced in the later version of the application were reported only rarely and may warrant reconsideration regarding their inclusion in the EMA protocol. Although tooth‐grinding behaviour was also reported infrequently, previous research indicates that it can increase the likelihood of individuals exhibiting TMD‐related pain symptoms [19], suggesting that it should remain a target for continued investigation.

Sensitivity analyses demonstrated highly consistent stabilization estimates across cohorts for relaxed jaw condition, jaw bracing, teeth contact, and clenching. Grinding was the only behaviour showing a somewhat different stabilization pattern. Because grinding is an infrequent behaviour, reliability estimates are particularly sensitive to sample size and behavioural classification. Nevertheless, the overall conclusions of the study remained unchanged, as four of the five core behaviours consistently achieved stable estimates within 2–3 monitoring days across cohorts.

Considering reliability gains, sample retention, and practical interpretability, a compliance threshold of twelve answered alerts per day (T ≥ 12) represented a reasonable balance between data quality and participant retention. Although ICC(A,1) values at the lowest compliance threshold (T = 1) fell within the “good” range, this threshold was not considered adequate for defining compliant data. First, the associated 95% confidence intervals were substantially wider, particularly for low‐frequency behaviours such as grinding, indicating imprecise and unstable reliability estimates. Second, one response per day does not meet the conceptual requirements of EMA, which relies on multiple daily observations to capture real‐time behavioural fluctuations. Third, ICC values at T = 1 are partly driven by the large sample size (*n* = 327), which can artificially stabilize between‐person variance and mask measurement error, thereby overstating reliability.

For these reasons, T = 1 was deemed statistically and methodologically insufficient, and higher thresholds were evaluated to identify a more reliable and clinically defensible criterion. Although reliability improved as the daily compliance threshold increased, several lower thresholds were also not suitable for defining compliant data. At T = 8, reliability improved but ICCs were still not consistently excellent across all behaviours, and some estimates remained borderline. Even at T = 10, reliability had not yet plateaued, with further gains in both ICC magnitude and confidence interval narrowing observed at higher thresholds. Collectively, these findings indicated that lower thresholds did not provide the combination of excellent reliability, precision, and methodological robustness required for a defensible standard. Therefore, T ≥ 12 represented the most appropriate and empirically supported compliance criterion for future EMA‐based assessment of awake bruxism. The mean daily response count in the pooled sample (14.3 ± 1.8 answered alerts per day) indicated that most participants naturally met this requirement without additional prompting, supporting the finding of Colonna et al. [20].

The large general‐population sample (*n* = 321) and the substantial volume of EMA reports (over 32 000) support the generalizability of these findings beyond clinical TMD cohorts and underscore the efficiency of EMA for assessing awake bruxism. The findings have clear clinical and practical relevance, demonstrating that a shorter, 3‐day EMA protocol, with ≥ 12 answered alerts per day, can yield reliable estimates of AB behaviours. This reduction in monitoring burden has the potential to improve compliance, broaden applicability in general‐population settings, and facilitate integration of EMA into routine clinical and research workflows whenever needed.

The present results pertain specifically to the use of BruxApp as an EMA tool and should not be extrapolated to its application within an Ecological Momentary Intervention (EMI) framework. Although fewer assessment days may enhance feasibility and adherence without materially affecting the reliability of awake bruxism monitoring, this consideration does not necessarily apply to intervention protocols.

EMI effectiveness depends on sustained awareness, behavioural change, and habit consolidation, yet the optimal duration, frequency, and intensity of BruxApp‐based EMI remain insufficiently defined. The impact of reducing intervention days on clinical outcomes is likewise unknown. Consequently, while abbreviated schedules may be adequate for EMA, longer and more persistent EMI protocols may be required to achieve stable behavioural modification. When EMA is intentionally applied as an EMI‐biofeedback intervention for TMD‐pain patients, extended monitoring durations can be implemented according to individual treatment requirements.

### 4.1 Limitations

The pooled sample was relatively large and drawn from three independent cohorts, and all participants were individuals who agreed to complete a 7‐day EMA protocol with a minimum of 12 alerts per day. This self‐selection may have introduced a compliance bias, as individuals who are more motivated, technologically comfortable, or symptom‐aware may be overrepresented. Second, although the five core awake bruxism behaviours were consistently assessed across cohorts, the additional behaviours introduced in later app versions were rarely endorsed, limiting the ability to evaluate their psychometric properties or clinical relevance. Future studies should reassess whether these low‐frequency behaviours warrant continued inclusion in EMA protocols. Finally, the analysis focused on temporal stability and compliance thresholds but did not examine potential moderators such as age, sex, psychological distress, or TMD related pain. These factors may influence both EMA engagement and AB expression, and their role should be explored in future work. It is also important to consider that psychological elements such as stress, anxiety, adherence, and reactivity to the monitoring process could have shaped the observed outcomes.

## 5 Conclusions

A three‐day EMA protocol requiring 12 completed alerts per day provides reliable estimates of the five core awake bruxism behaviours. These findings support a 57% reduction in monitoring burden compared with conventional 7‐day protocols and may facilitate broader implementation of EMA in both research and clinical settings.

## Author Contributions

**Alona Emodi‐Perlman:** conceptualization, funding acquisition, methodology, writing – review and editing, validation, visualization, supervision, writing – original draft, formal analysis. **Ilana Eli:** conceptualization, funding acquisition, writing – original draft, methodology, validation, visualization, writing – review and editing, formal analysis, supervision. **Anna‐Yael Czygrinow:** conceptualization, investigation, data curation. **Lihi Keren:** investigation, conceptualization, data curation. **Khalil Masarwe:** conceptualization, investigation, data curation. **Noa Ventura:** conceptualization, investigation, data curation.

## Funding

The study was funded by Grant No. 3737/24 of the Israel Science Foundation.

## Conflicts of Interest

The authors declare no conflicts of interest.

## Supporting information


**Table S1:** Participant‐level 7‐day percentages for 10 behaviours (*N* = 221).
**Table S2:** ICC(A,1) with 95% CI between cumulative L‐day mean and 7‐day gold standard, by behaviour (*N* = 327)*.
**Table S3:**: Cohort specific sensitivity analysis.

## Data Availability

The data that support the findings of this study are available on request from the corresponding author. The data are not publicly available due to privacy or ethical restrictions.
