# Supplementary material for: Towards Optimized Awake Bruxism Metrics: Empirical Definition of Minimal Ecological Momentary Assessment Requirements
Source: J Oral Rehabil. 2026 Jul 9;53(11):2112–9. doi: 10.1111/joor.70263 (PMC13653295; doi:10.1111/joor.70263)
Supplement: Supplementary file 1 [file JOOR-53-2112-s001.docx]

**Toward Optimized Awake Bruxism Metrics: Empirical Definition of Minimal Ecological Momentary Assessment Requierements - Supplementary Material**

**Table S1:** Participant-level 7-day percentages for 10 behaviors (N=221).

| **Behavior** | **Mean % (SD)** | **Median % [IQR]** |
| --- | --- | --- |
| Relaxed | 48.52 (25.94) | 48.57 [28.54-68.74] |
| Bracing | 14.89 (15.44) | 10.17 [3.39-21.94] |
| Teeth contact | 17.84 (13.83) | 15.58 [6.59-25.87] |
| Clenching | 7.49 (11.26) | 2.99 [0.95-9.89] |
| Grinding | 1.72 (4.52) | 0.00 [0.00-1.10] |
| Push tongue | 1.21 (2.82) | 0.00 [0.00-1.10] |
| Place tongue between teeth | 2.17 (5.58) | 0.00 [0.00-1.79] |
| Bite/chew/play tongue-cheeks-lips | 3.00 (5.26) | 0.89 [0.00-3.95] |
| Hold/bite objects | 0.50 (1.81) | 0.00 [0.00-0.00] |
| Chewing gum | 2.65 (7.82) | 0.00 [0.00-2.38] |

**Table S2:** ICC(A,1) with 95% CI between cumulative L-day mean and 7-day gold standard, by behavior (N=327)*

| **Behavior** | **L= 1** | **L= 2** | **L= 3** | **L= 4** | **L= 5** | **L= 6** | **L=7**** |
| --- | --- | --- | --- | --- | --- | --- | --- |
| Relaxed jaw posture | 0.628 (0.546-0.695) | 0.740 (0.675-0.795) | **0.885 (0.854-0.910)** | 0.949 (0.936-0.960) | 0.979 (0.974-0.983) | 0.994 (0.993-0.995) | 1.00 |
| Jaw bracing | 0.654 (0.576-0.725) | 0.715 (0.636-0.782) | **0.860 (0.814-0.895)** | 0.938 (0.916-0.954) | 0.975 (0.966-0.981) | 0.994 (0.991-0.995) | 1.00 |
| Teeth contact | 0.500 (0.394-0.590) | 0.756 (0.697-0.807) | **0.874 (0.840-0.901)** | 0.932 (0.911-0.948) | 0.967 (0.957-0.975) | 0.990 (0.987-0.992) | 1.00 |
| Clenching | 0.490 (0.360-0.608) | 0.775 (0.690-0.842) | **0.887 (0.841-0.923)** | 0.943 (0.918-0.960) | 0.974 (0.961-0.982) | 0.993 (0.989-0.995) | 1.00 |
| Grinding | 0.550 (0.346-0.759) | **0.806 (0.620-0.909)** | 0.863 (0.720-0.935) | 0.908 (0.800-0.957) | 0.952 (0.879-0.980) | 0.982 (0.957-0.993) | 1.00 |

** ICC(A,1) = intraclass correlation coefficient, two-way random effects, absolute agreement, single measures. Bold values indicate the first window reaching ICC ≥ 0.80 (minimum stable monitoring duration). L = 7 equals 1.000 by definition because both vectors are identical.*

**** *Values are ICC(A,1) with 95% CI. L=7 equals 1.000 by definition because the compared estimates are identical.*

**Table S3**: Cohort specific sensitivity analysis

| **Cohort*** | **Behavior** | **Minimum days to ICC >= 0.80** | **ICC at threshold (95% CI)** | **N** |
| --- | --- | --- | --- | --- |
| Pooled (S1+S2+S3) | Relaxed jaw posture | 3 | 0.885 (0.854-0.908) | 327 |
| Pooled (S1+S2+S3) | Jaw bracing | 3 | 0.860 (0.816-0.895) | 327 |
| Pooled (S1+S2+S3) | Teeth contact | 3 | 0.874 (0.840-0.902) | 327 |
| Pooled (S1+S2+S3) | Clenching | 3 | 0.887 (0.844-0.923) | 327 |
| Pooled (S1+S2+S3) | Grinding | 2 | 0.806 (0.607-0.905) | 327 |
| S1 only | Relaxed jaw posture | 2 | 0.896 (0.843-0.931) | 106 |
| S1 only | Jaw bracing | 2 | 0.861 (0.803-0.911) | 106 |
| S1 only | Teeth contact | 2 | 0.813 (0.743-0.872) | 106 |
| S1 only | Clenching | 2 | 0.858 (0.802-0.913) | 106 |
| S1 only | Grinding | 6 | 0.925 (0.904-0.985) | 106 |
| S2+S3 | Relaxed jaw posture | 3 | 0.855 (0.811-0.892) | 221 |
| S2+S3 | Jaw bracing | 3 | 0.837 (0.767-0.889) | 221 |
| S2+S3 | Teeth contact | 3 | 0.870 (0.826-0.904) | 221 |
| S2+S3 | Clenching | 3 | 0.892 (0.825-0.933) | 221 |
| S2+S3 | Grinding | 2 | 0.836 (0.656-0.922) | 221 |

** S1 included the five-behavior app version. S2+S3 included the ten-behavior app version. The analysis was restricted to the five core behaviors common to all cohorts*.
